# Extraction, Purification, Physicochemical Properties, and Activity of a New Polysaccharide From *Cordyceps cicadae*

**DOI:** 10.3389/fnut.2022.911310

**Published:** 2022-06-09

**Authors:** Zizhong Tang, Wenjie Lin, Yusheng Chen, Shiling Feng, Yihan Qin, Yirong Xiao, Hong Chen, Yuntao Liu, Hui Chen, Tongliang Bu, Qinfeng Li, Yi Cai, Huipeng Yao, Chunbang Ding

**Affiliations:** ^1^College of Life Sciences, Sichuan Agricultural University, Ya’an, China; ^2^Sichuan Agricultural University Hospital, Sichuan Agricultural University, Ya’an, China; ^3^College of Food Science, Sichuan Agricultural University, Ya’an, China

**Keywords:** physicochemical properties, polysaccharide, *Cordyceps cicadae* (*C. cicadae*), extraction, purification

## Abstract

The polysaccharides from *C. cicadae* were extracted by ultrasonically-assisted enzymatic extraction (UAEE). Response surface analysis was used to determine the optimum parameters as follows: addition of enzymes, 0.71%; extraction temperature, 60°C; extraction time, 18 min; liquid-solid ratio, 46:1 (mL/g). The extraction yield of polysaccharide was 3.66 ± 0.87%. A novel polysaccharide fraction (JCH-a1) from *C. cicadae* was extracted and then purified by cellulose DEAE-32 and Sephadex G-100 anion exchange chromatography. The analysis results showed that the molar ratio of galactose, glucose, and mannose in JCH-a1 cells (60.7 kDa) was 0.89:1:0.39. JCH-a1 with a triple helix contains more α-glycosides and has strong thermal stability. Moreover, JCH-a1 showed strong antioxidant activity and acted as a strong inhibitor of α-glucosidase *in vitro*. In addition, JCH-a1 can prolong the lifespan of *C. elegans*. The present study might provide a basis for further study of JCH-a1 as an antioxidant and hypoglycemic food or drug.

## Introduction

*C. cicadae* is an entomogenous fungus, and its cluster is formed after the larvae were infected with the fungus *Isaria cicadae miquel* of the ergot family (1). For centuries, as a medicinal and edible fungus, *C. cicadae* have been used as food, tonic, and folk medicine to treat malaria, palpitations, diabetes, eye diseases, dizziness, and chronic kidney diseases (2). It contains nucleoside, ergosterol, amino acid, polysaccharide, mannitol, and several other chemical components (3). In previous studies, polysaccharides from *C. cicadae* were found to be the main active ingredient of its antitumor, immunomodulatory, renal protective, antioxidative, and antibacterial activity (2). It was found that *C. cicadae* polysaccharides had strong free radical scavenging ability and significant antioxidant activity *in vitro* (4). In addition, it could significantly improve the survival rate of PC12 cells after glutamate-induced oxidative damage (5). *In vivo* antioxidant activity of *C. cicadae* polysaccharides has been reported in *Drosophila melanogaster* (6). However, whether it has antioxidant activity in *Caenorhabditis elegans* has not been cleared. Therefore, based on the antioxidant assays *in vitro*, this study further explored the antioxidant activity of *C. cicadae* polysaccharide in *C. elegans*.

It is worth noting that the existing studies mainly reported the bioactivity of *C. cicadae* polysaccharide, but there were few studies on its extraction methods. The extraction method can not only significantly affect the content and yield of polysaccharide, but also significantly affect its structural characteristics and biological activity (7). Ultrasonic extraction is an effective method to destroy cell wall and improve mass transfer efficiency, while enzyme can degrade cell wall and effectively promote the release of intracellular polysaccharides (8). So far, ultrasonic assisted enzyme extraction (UAEE) has been used to separate polysaccharides from various natural organisms (9, 10). However, UAEE has not been used to extract polysaccharides from *C. cicadae*, so the importance of this study has been established. Response surface method (RSM) is an effective statistical technique, which is usually used to optimize complex process conditions. The main advantage of RSM is to reduce the number of experiments required to evaluate multiple parameters, their interactions and generate mathematical models to determine the optimal value (11). Therefore, multiple independent variables and their interaction methods were optimized by RSM to improve the efficiency of UAEE in *C. cicadae* polysaccharide.

In recent years, many reports have shown that the biological activities and structures of polysaccharides are closely related (12). Li et al. reported that the antioxidant activity was related to the uronic acid content (13). 1,3-β-D-glucan from *Lentinus edodes* was found to show strong anti-cancer activity (14). Polysaccharides with a single side chain have been revealed to promote cytokine release (15). Kiho T found that the phagocytosis capability of *C. cicadae* polysaccharides was related to their content of mannose residues (16). However, the structure-activity relationship of polysaccharides in *C. cicadae* has not been studied extensively.

Based on this, this study first optimized the process parameters of UAEE assisted extraction of *C. cicade* crude polysaccharide (JCH) by RSM method. Furthermore, the physical and chemical properties, monosaccharide composition, molecular weight, and structural characteristics of the purified components were determined by multispectral technology. Subsequently, we evaluated the free radical scavenging and inhibition α-glucosidase activity of polysaccharides *in vitro*. In addition, the lifespan, enzyme activity and stress resistance of polysaccharides to *C. elegans* were also studied to explore their antioxidant activity *in vivo*. This study provides a basis for clarifying the structure-activity relationship of *C. cicadae* polysaccharide, and shows its potential as a new antioxidant and hypoglycemic in functional food and pharmaceutical industry.

## Materials and Methods

### Chemicals and Reagents

Bovine Serum Albumin (BSA), 2, 2-Diphenyl-1-picrylhydrazyl (DPPH) and 2, 2′-azino-bis (3-ethylbenzthiazoline-6-sulfonic acid) (ABTS) were purchased from Sigma-Aldrich Corporation (United States). V_C_ (Vitamin C, AR) was obtained from Sinopharm Chemical Reagent Co. (Shanghai, P. R. China). Unless otherwise stated, all reagents used were of analytical grade.

### Materials

Fresh *C. cicadae* fungi were picked from Wufengshan Park, Dazhu, Sichuan Province, China (107° 16′ 17″∼107° 25′ 34″ E, 30° 43′ 34″∼30° 51′ 06″ N. Altitude: 600–1,080 m. Annual total precipitation: 1202.9 mm. Mean daily temperature: 16.7°C. Maximum humidity: 99%), identified by Professor Chen Hui, Sichuan Agricultural University. The freshly harvested samples were dried at 60°C and ground into powders for JCH extraction.

### Ultrasonic-Assisted Enzymatic Extraction of Polysaccharides

#### Extraction Method

Ultrasonic assisted enzyme complex enzyme (cellulase: chitinase = 1:1, w/w) was extracted with ultrasonic cleaner (Shengzheng JATO Science Technologies, Co., Ltd., Shengzheng, China). The *C. cicadae* powder (1.0 g) was placed into a beaker and distilled water was added (15:1–55:1 mL/g) as well as complex enzyme (the amount of enzyme was 0.2 ∼ 1%). Then it was extracted in an ultrasonic cleaning machine (ultrasonic power 240 W) at 30–50°C for 10–50 min. The extract was concentrated, precipitated with 95% ethanol, centrifuged (8,000 rpm for 20 min) and collected. Crude polysaccharide (JCH) was obtained after freeze drying.

#### Response Surface Method Experimental Design

On the basis of single factor experiment, the response surface experiment was designed by Box-Behnken. Taking the extraction rate as the evaluation index and the amount of enzyme, ultrasonic temperature, ultrasonic time, and solid-liquid ratio as the investigation factors, the extraction process of polysaccharide was optimized 4.

#### JCH Purification

JCH was deproteinized with Sevage (17). Then, distilled water and NaCl solution with different concentrations (0.1, 0.3, 0.5, 0.8 M) were used to pass through DEAE-32 column (3 × 50 cm) at a rate of 1 mL/min. A total of 10 mL eluate was collected per tube and polysaccharide content was determined by phenol sulfuric acid (18).

The homogeneous fractions after dissolution in distilled water were collected and named JCH-a. Next, the dissolved JCH-a was eluted from the Sephadex G-100 (25 mm × 50 cm) column with distilled water at a flow rate of 0.5 mL/min. Collected 8 mL eluent from each test tube and measured the sugar content in each tube by phenol sulfuric acid method (18). Subsequently, JCH-a was collected, dialyzed (8,000–14,000 Da), and lyophilized to obtain purified *C. cicadae* polysaccharides, which are denoted JCH-a1 here.

### Physicochemical Properties

#### Major Chemical Content

The contents of neutral sugar and protein were determined by phenol sulfuric acid method (18) and Bradford method (19), respectively.

#### Molecular Weights

The molecular weights of polysaccharides were measured by gel permeation chromatography (GPC) with an LC20 high-performance liquid chromatography pump (Shimadzu, Japan) and an RID-20 refractive index detector (Shimadzu, Japan). The column was eluted with an aqueous solution of sodium nitrate (0.1 M) at a flow rate of 0.5 mL/min. The molecular weight of JCH-a1 was calculated from curves calibrated with T-series dextran standards (MW 642, 334, 49.4, 2.2, 0.63 kDa).

#### Monosaccharide Composition

The monosaccharide composition of JCH-a1 was determined by HPLC (LC-20AD, Japan) with a C18 Xtimate column (4.6*200 mm 5 μm). The column temperature was set to 30°C, the flow rate was set to 1 mL/min, the injection volume was 20 μL, and the mobile phase was 0.05 M 83% potassium dihydrogen phosphate solution (pH 6.70 with sodium hydroxide solution) to 17% acetonitrile. Mannose, ribose, rhamnose, glucuronic acid, galacturonic acid, N-acetyl-glucosamine, glucose, N-acetyl-galactose, galactose, xylose, arabinose, and fucose were chosen as standards.

#### FT-IR Spectrometry

Dried polysaccharides (2 mg) were mixed with KBr (200 mg) and observed with FT-IR spectroscopy (Nicolet 6700, Thermo Scientific, America) at 4,000–400 cm^–1^.

#### Scanning Electron Microscopy

JCH-a1 was fixed on the copper platform with conductive adhesive and sprayed with gold powder. Scanning Electron Microscopy (SEM) images of JCH-a1 at different magnification were obtained by field emission scanning electron microscope (Hitachi su8220, Japan).

#### Atomic Force Microscopy

After the polysaccharide was completely dissolved in distilled water (10 μg/mL), with 0.22 μm membrane filtration, 10 μL dilution droplets were placed on the surface of newly cracked mica and dried overnight at room temperature. The molecular morphology of the polysaccharide was observed directly using a multimode 8 atomic force microscope (Dimension Icon, Germany).

#### Differential Scanning Calorimetric Analysis

The thermal properties of the polysaccharides were evaluated by differential scanning calorimetry (DSC). In the DSC (TA instruments Q 20-DSC, America) test, 2 mg of dried sample was placed in an aluminum pan. Then, the polysaccharides were scanned and heated from 30 to 300°C at a heating rate of 10°C/min under a N_2_ atmosphere.

#### Helix-Coil Transition Assay

The polysaccharide solution (2 mg/mL) and Congo red solution (0.2 mM) were mixed at a volume ratio of 1:1, and the maximum absorption wavelengths λ_max_ of the mixed solution in a NaOH solution of 0–0.4 mol/L were measured successively. The λ_max_ curves were drawn with the final concentration of NaOH of each mixed system as the abscission and λ_max_ as the ordinate.

#### Crystallinity

The crystallinity was observed by X-ray diffraction (XRD) (Brucker D8 Advance, Germany) at scattering angles of 2θ = 10°∼ 80°, a step size of 0.05, and an exposure time of 1 s.

#### Nuclear Magnetic Resonance Analyses

Samples (80 mg) were dissolved in 2 mL D_2_O and freeze dried, repeated three times. The Nuclear Magnetic Resonance (NMR) spectra of ^1^H and ^13^C were obtained on a Bruker 600 MHz NMR spectrometer (Bruker Biospin, Milton, Canada).

#### Antioxidant Activities

The DPPH, ABTS, hydroxyl (•OH), and superoxide (•O_2_^–^) radical-scavenging activity of polysaccharides were measured following previously described method by Wang et al. (20), Zhang et al. (21), Ren et al. (22), and Ma et al. (23).

#### Inhibition of α-Glucosidase *in vitro*

The samples’ inhibitory effect on α-glucosidase and kinetics of inhibition were studied following a previous method (24, 25).

#### Strains and Culture

*C. elegans* N2 and *E. coli* OP50 were obtained from botany laboratory, Sichuan Agricultural University. *C. elegans* were grown on NGM plates, inoculated with *E. coli* OP50 at 20°C.

#### Food Clearance Assay

Polyaccharide concentrations were screened according to Voisine (26), estimated as the rate of *E. coli* consumption. Synchronized nematodes (L4) approximately 20–30 were transferred to 96-well plates containing different concentrations of polysaccharide (0, 0.1, 0.5, 1.0, 2, 5 mg/mL) and grown at 20°C. The absorbance at 598 nm was measured daily.

#### Lifespan Assay

Synchronous L1 larvae were cultured in NGM for 3 days and reached L4 stage. The synchronized nematodes (L4) were transferred to NGM containing polysaccharide (0.1, 0.3, 0.5 mg/mL) and without polysaccharide (control). The number of nematodes was counted every day and transferred to a new NGM board (add polysaccharide) in the first 7 days, to rule out the influence of their offspring, until all the nematodes died. *C. elegans* was judged dead if there was no response to platinum wire contact.

#### Lifespan Under Different Stressors

The worm culture of stress assays was the same as the previous ordinary lifespan assay. After 5 days, nematodes were transferred to new NGM plates and grown at 37°C for thermal stress experiments. Under UV irradiation, UV stress experiments were performed. The NGM of osmotic, oxidative stress analysis contains 300 mM NaCl and 30 mM H_2_O_2_, respectively. Then, the survival was monitored hourly, until all worms died.

#### Determination of Antioxidant System

After the nematode was cultured to L4 stage, superoxide dismutase (SOD) activity, catalase (CAT) activity, glutathione peroxidase (GSH Px) and Malonaldehyde (MDA) levels were measured, and the results were consistent with the instructions on the kit. These tests were repeated three times.

### Statistical Analysis

The data from three independent experiments are presented as the means ± *SD*. SPSS 19.0 was utilized as the software to analyze the data, which were subsequently processed with Excel 2010. The results of the response surface experiments were analyzed by Design-Expert 12.0 software.

## Results

### Optimization Analysis

The results of single factor experiment showed that the addition of enzyme 0.6%, temperature 60°C, time 20 min and solid-liquid ratio of 1:45 g/mL were selected to the next step of optimization (Supplementary Figure 1). The optimized range of response surface factors for the single factor screen was shown in Supplementary Table 1. The experimental results obtained from the Box-Behnken design (BBD) was shown in Supplementary Table 2. The quadratic polynomial regression equation of the response surface model was obtained by ANOVA, which can be expressed as follows:


$$\begin{array}{c} Y\left(\%\right)=3.65-0.2A-0.0217B-0.1375C+0.2225D\\ \;-0.0950\mathrm{AB}-0.0275\mathrm{AC}-0.3025\mathrm{AD}-0.3700\mathrm{BC}\\ \;+0.0350\mathrm{BD}-0.0300\mathrm{CD}-0.3155A^{2}-0.2405\mathrm{B2}\\ \;-0.3817C^{2}-0.3343D^{2}. \end{array}$$

where Y is the extraction yield of JCH%, A is the enzyme addition, B is the ultrasonic temperature, C is the ultrasonic time, and D is the liquid-solid ratio.

Analysis of variance (ANOVA) was used to test the adequacy and suitability of the established model. The results of ANOVA of RSM are shown in Supplementary Table 3, including *F*, *P*-value, sum of squares, mean square, degree of freedom, and other parameters. A higher model *F*-value (22.69) and a lower *p*-value (< 0.0001) indicated that the regression equation could explain most of the variation in response. In addition, statistical significance was established for the validity of the model when *P*-value < 0.05. These results show that the model was very significant. The fitting degree of the model was evaluated by the determination coefficient (*R*^2^) and adjustment coefficient (*R*^2^_*adj*_), which were calculated as 0.9578 and 0.9156, respectively.

Therefore, the model was verified to be well fitted, and the experimental and predicted values were highly correlated. In summary, the regression model for JCH was satisfactory. Moreover, enzyme addition (A), ultrasonication time (B), and liquid–solid ratio (D) played a significant (*P* < 0.001) role. The amount of enzyme addition vs. liquid–solid ratio (AD) and ultrasonic temperature vs. ultrasonic time (BC) were all found to be highly significant (*P* < 0.0001).

**FIGURE 1:**
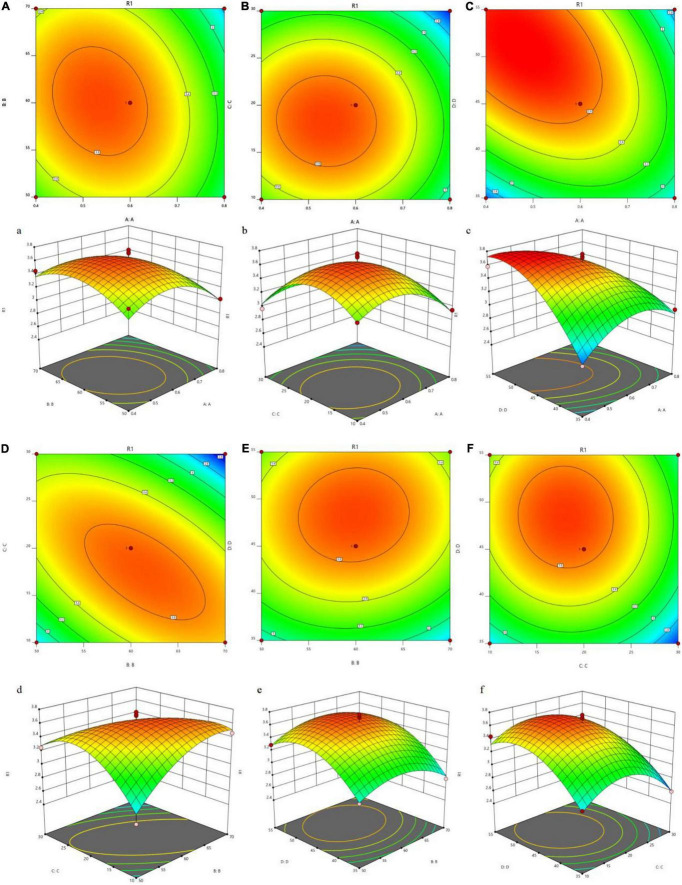
Tri-dimensional response surface plots and contour plots illustrating the interaction between the different variables on the yield of JCH. (**(a,A)** enzymatic addition and ultrasonic temperature; **(b,B)** enzymatic addition and ultrasound time; **(c,C)** enzymatic addition and liquid-solid ratio; **(d,D)** ultrasonic temperature and ultrasonic time; **(e,E)** ultrasonic temperature and liquid-solid ratio; **(f,F)** ultrasonic time and liquid-solid ratio; R1, JCH yield).

For investigations on the variable interactions to obtain the optimal level of each variable, 3D response surfaces (Figure 1) were created by Design Expert 12. The response surface of the model can illustrate the interactions among variables and provide information on the optimal conditions for polysaccharide extraction. It can be inferred from Figure 1 that the optimal extraction conditions for JCH were enzyme addition of 0.71%, 60°C, 18 min, and a liquid–solid ratio (46 mL/g). The predicted extraction rate under these conditions was 3.45%.

### Verification of the Predictive Model

The applicability of the model was tested under the conditions of enzyme addition (0.71%), ultrasonic temperature (60°C), ultrasonic time (18 min) and liquid-solid ratio (46 mL/g). The final average value obtained from the actual experiment (3.66 ± 0.87% (*n* = 3)) was similar to the theoretical value (3.45%), indicating that the model was available. According from Supplementary Table 4, enzyme-assisted extraction (EAE), ultrasound-assisted extraction (UAE) and UAEE were compared. At the same material liquid ratio and extraction time, the polysaccharide yields of EAE and UAE were 1.73 ± 0.69% and 1.54 ± 0.46%, respectively. Obviously, UAEE was more effective than UAE and EAE.

### JCH Purification

**FIGURE 2:**
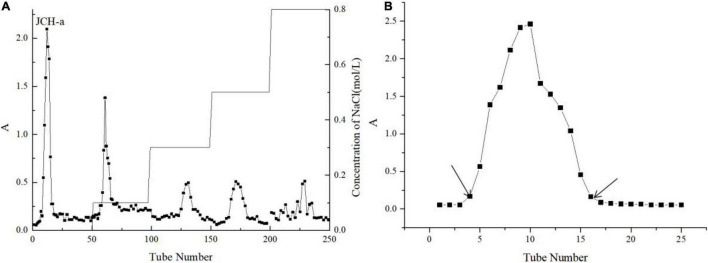
**(A)** The crude JCH was applied to a DEAE–32 column **(B)** JCH-a obtained from DEAE–32 was eluted with a Superdex-G100.

JCH was separated by a DEAE-32 cellulose column with deionized water, and the principal component of the eluent was named JCH-a (Figure 2A). A Sephadex G-100 column was used to further separate JCH-a, producing a fine polysaccharide fraction named JCH-a1 after concentration, dialysis, and lyophilization (Figure 2B). The total sugar content of JCH-a1 was 90.30%, and JCH-a1 also contained a small amount of protein (2.32%).

### Monosaccharide Compositions and Molecular Weight

**FIGURE 3:**
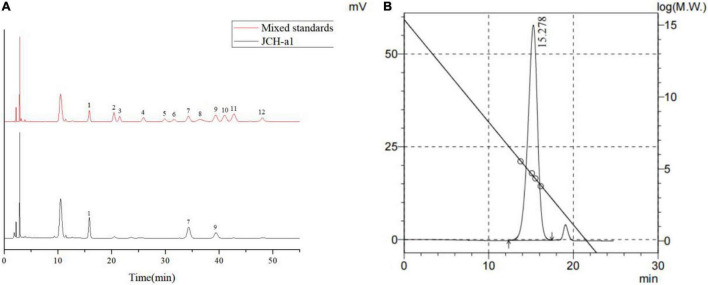
**(A)** HPLC chromatogram of the monosaccharide standards and JCH-a1. Peaks: 1 Mannose, 2 Ribose, 3 Rhamnose, 4 Glucuronic acid, 5 Galacturonic acid, 6 N- acetyl-glucosamine, 7 Glucose, 8 N- acetyl-galactose, 9 Galactose, 10 Xylose, 11 Arabinose, 12 Fucose; **(B)** GPC of JCH-a1.

The monosaccharide compositions of JCH-a1 were measured by high-performance liquid chromatography (HPLC), and the results are shown in Figure 3A. Judging from the figure, JCH-a1 is composed mainly of galactose, glucose, and mannose with a molar ratio of 0.89:1:0.39. According to the GPC results (Figure 3B), the average molecular weight of JCH-a1 is 60.7 kDa.

### Fourier Transform Infrared Spectral Analyses

The Fourier Transform Infrared (FTIR) spectra of JCH-a1 are given in Supplementary Figure 2, in which wide peaks are seen at 3,361 cm^–1^. This signal is attributed to the stretching vibration of −OH. The signals at approximately 2,900 cm^–1^ were attributed to C-H stretching vibrations, and the strong absorption peaks at 1,639 cm^–1^ were signals of C = O stretching vibrations. The C-H variable angular vibration was reflected as peaks at 1,400 cm^–1^ (27). Absorption peaks at these four frequencies are characteristic of polysaccharides. The band of 1,000–1,200 cm^–1^ was the bending or tensile vibration of C-O groups. The absorption peak at 879 cm^–1^ shows that JCH-a1 contains β-type glycosidic bonds, and there are many subtle absorption peaks at 855–810 cm^–1^, suggesting a larger component of α-type glycosidic bonds than β-type glycosidic bonds (28).

### Surface Topography

**FIGURE 4:**
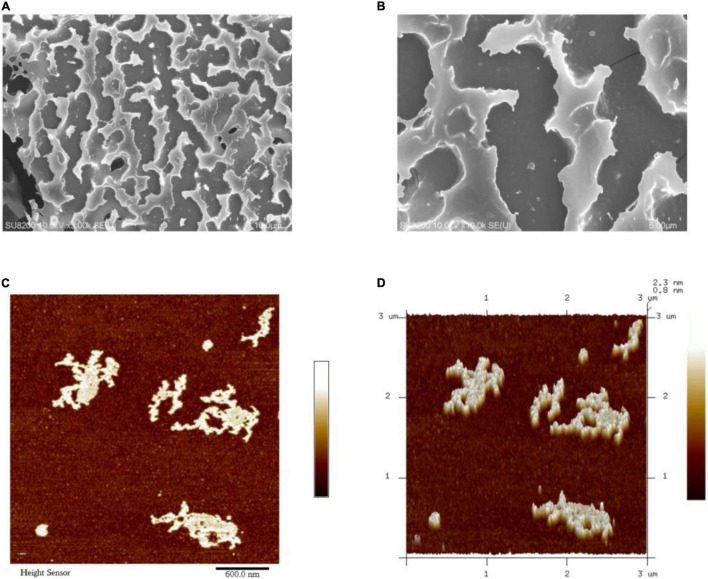
SEM of JCH-a1 [**(A)** 10 μm; **(B)** 5 μm]; AFM images of JCH-a1 **(C,D)**.

The surface morphology of JCH-a1 was observed by SEM, and the results are shown in Figures 4A,B. The figures show that the surface of JCH-a1 is rough and furrowed, suggesting excellent rehydration properties due to the higher content of branches (29). The Atomic Force Microscopy (AFM) analysis results of JCH-a1 are shown in Figures 4C,D. JCH-a1 was found to form large lumps, indicating that the molecules were aggregated, branched, and entangled in their structures. Generally, the diameter of polysaccharide single chains is 0.1–1 nm, which is much smaller by an order of magnitude than the diameter of JCH-a1 observed by AFM. Therefore, JCH-a1 is not a molecule of single sugar chains. This phenomenon could be attributed to the large number of hydroxyl groups in the polysaccharide chain, which facilitate the formation of intermolecular or intramolecular hydrogen bonds, thereby intertwining the sugar chains, and creating irregular structures with the help of van der Waals forces (30).

### Differential Scanning Chromatography Analyses

With DSC analysis, mass changes in samples due to dehydration, decomposition, and oxidation can be quantitatively measured with respect to time and temperature (31). A broad endothermic peak at approximately 62.6°C (Supplementary Figure 3) can be explained by the loss of free or bound water in the polysaccharide. Moreover, Supplementary Figure 3 shows that the glass transition temperature (Tg) of JCH-a1 may be above 300°C. Therefore, the gum would be stable at normal operational temperatures (32).

### Helix-Coil Transition Assays

**FIGURE 5:**
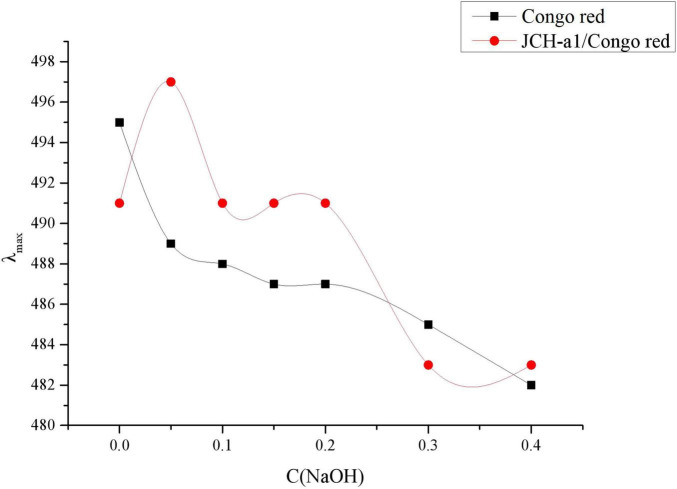
Changes in the maximum absorption wavelength (λ_max_) of JCH-a1/Congo red complex with various NaOH concentrations.

Congo red is an acidic dye capable of forming complexes with polysaccharides in a triple-helix conformation (33). The results of Congo red analysis of JCH-a1 are displayed in Figure 5. The maximum wavelengths of JCH-a1/Congo red varied with different NaOH concentrations. the maximum wavelengths of JCH-a1/Congo red at 0.05 M NaOH showed significant redshifts from 489 to 497 nm compared with the control group. With higher NaOH concentrations, The wavelength of the experimental groups dropped gradually, suggesting that JCH-a1 had a triple helix structure.

### Crystallinity Analyses

Diffraction techniques are important to determine the crystal structure and predict other physical properties of polysaccharides, including the swelling properties, flexibility, tensile strength, and solubility (34). Supplementary Figure 4 shows the XRD pattern of JCH-a1. When the diffraction angle (2θ) was 3∼80°, JCH-a1 showed only a dispersion peak at approximately 2θ = 20°, and no strong absorption peak appeared. This result is consistent with the X-ray powder diffraction (XRD) results of other polysaccharides with a very low content of neutral or uronic acids (35). Therefore, the polysaccharide component of JCH-a1 is verified to be amorphous with some microcrystalline structures but without the formation of single crystals, which is manifested by its good solubility in water.

### Nuclear Magnetic Resonance Analysis

The structure of JCH-a1 was further analyzed using NMR techniques. ^1^H NMR profiles showed that the chemical shift of JCH-a1 was mainly between 3.5 and 5.3 ppm. It can be seen from Supplementary Figure 5A, the signals in the range of 4.8–5.3 ppm in the ^1^H NMR spectrum indicated that JCH-a1 contained a α-glycoside structure and small β-type glycosidic bonds (36). These results were consistent with the glycoside bond types of FT-IR analysis. The weak peaks at 5.32 ppm were attributed to α-Gal residues (37), and the peak at 4.90 ppm was attributed to α-Glc residue. As shown in the ^13^C NMR spectrum (Supplementary Figure 5B), multiple isopropanol carbon signals existed in the 100.68 ppm range, while other non-isopropanol carbon signals occurred in the range of 60.7–80.9 ppm.

### Antioxidant Activity

In this study, JCH and JCH-a1 were investigated for their scavenging activities of DPPH, ABTS, •OH, and •O_2_^–^, and the results were shown in Supplementary Figure 6. The purple ethanol solution of DPPH has a strong absorption peak at 517 nm, which is a very stable free radical. If the antioxidant activity is strong, the purple color would fade, making it a good indicator of the antioxidant activity (38). The *IC*_50_ values of the DPPH radical scavenging activity of JCH and JCH-a1 were 0.67 and 1.225 mg/mL, respectively (Supplementary Figure 6A). For ABTS radicals, their color can be changed by electrons or hydrogen atoms provided by antioxidants (39). The *IC*_50_ values of JCH and JCH-a1 for ABTS radicals were 0.33 and 0.707 mg/mL, respectively (Supplementary Figure 6B). Excessive production of •OH and •O_2_^–^ groups would break the homeostasis and promote various diseases (40). The *IC*_50_ values of the scavenging activities of JCH and JCH-a1 to hydroxyl radicals were 0.507 and 1.546 mg/mL, respectively (Supplementary Figure 6C), and those two values became 1.045 and 1.644 mg/mL for superoxides, respectively (Supplementary Figure 6D). Judging from the antioxidant evaluation results, JCH, JCH-a1, and V_C_ tended to show higher scavenging activity for DPPH, ABTS, hydroxyl, and superoxide radicals at higher concentrations. In addition, the scavenging activity of JCH was better than the scavenging activity of JCH-a1. Therefore, crude polysaccharides are confirmed to exhibit good antioxidant activity *in vitro*, probably due to their higher protein contents. Similar studies have found that antioxidant polysaccharides are mostly crude or complexed with proteins and polyphenols (41).

### The Inhibitory Activity to α-Glucosidase

**FIGURE 6:**
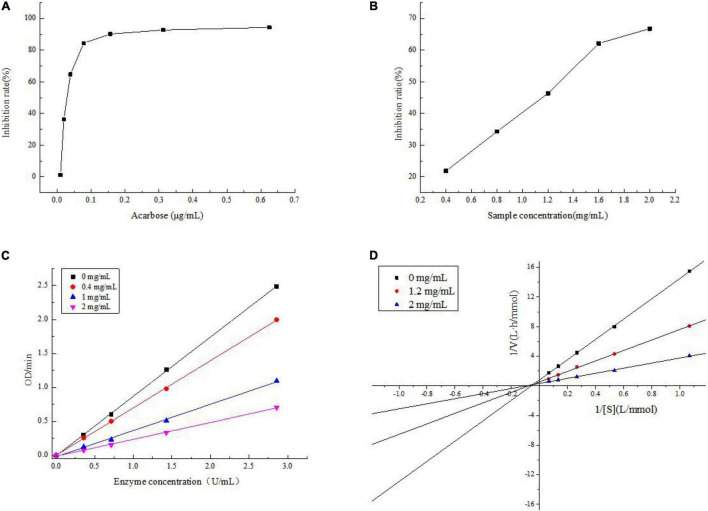
**(A)** Inhibition on the α-glucosidase activity of acarbose; **(B)** inhibition on the α-glucosidase activity of JCH-a1; **(C)** inhibition kinetic diagram of JCH-a1; **(D)** lineweaver-Burk plots for a-glucosidase inhibition by JCH-a1.

The inhibitory activities of acarbose and JCH-a1 against α-glucosidase are displayed in Figures 6A,B. When the polysaccharide concentration was 2 mg/mL, JCH had no inhibitory activity, while JCH-a1 showed an inhibition rate against α-glucosidase of 66.81%. The inhibitory activity was found to be concentration-dependent. The *IC*_50_ values of acarbose and JCH-a1 were 0.03 and 1.203 mg/mL, respectively. The inhibition kinetics diagram is given in Figure 6C. The concentrations of polysaccharides were 0, 0.4, 1.2, and 2 mg/mL, and the concentrations of the enzyme were plotted by absorbance (speed) per unit time. As the enzyme concentration rose, the curve slope dropped with an identical inhibitor concentration. All 4 curves passed through the origin. Therefore, the inhibition of α-glucosidase by JCH-a1 is reversible.

The inhibition kinetics of JCH-a1 were studied by Lineweaver-Burk plots. In Figure 6D, the X-axis represents the reciprocal of the matrix pNPG concentration, and the Y-axis represents 1/V calculated by absorbance. The double reciprocal curves of inhibitors with different concentrations intersect in the second quadrant, indicating that the maximal velocity (V_max_: the reciprocal of the y-intercept) decreased. On the other hand, after adding polysaccharide into the reaction system, the Michaelis constant (reciprocal of K_m_: X intercept) would increase, suggesting that upon the combination with the polysaccharides, the affinity of α-glucosidase to its substrate drops. Therefore, JCH-a1 inhibits α-glucosidase activity via a mixed-type mechanism (24).

### Polysaccharide Concentration Range

**FIGURE 7:**
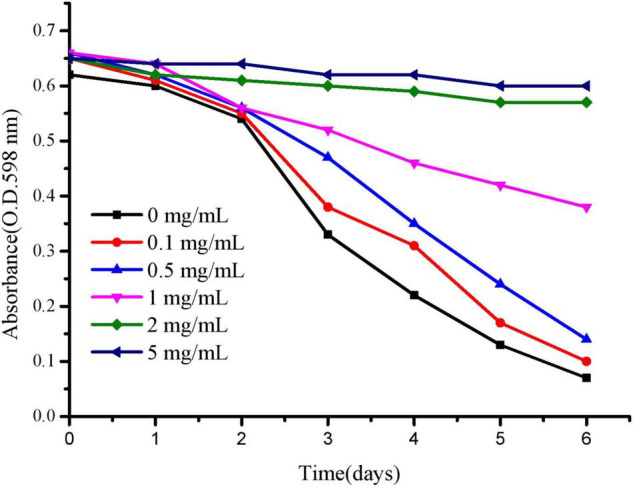
The OD of *E. coli* is reported daily for each concentration of JCH-a1.

To effectively evaluate the anti-aging and stress resistance of polysaccharides to *C. elegans*, we mainly selected the appropriate concentration of polysaccharides to culture *C. elegans*. As shown in Figure 7, *C. elegans* was exposed to 2–5 mg/mL polysaccharide, delaying food clearance. At 0–0.5 mg/mL, according to the absorbance change of the control group, the value after polysaccharide treatment decreased rapidly, indicating that the concentration was more suitable for the growth of worms. For polysaccharides, the optimal concentration of *C. elegans* was below 0.5 mg/mL, and the following tests were selected.

### Lifespan Analysis

**FIGURE 8:**
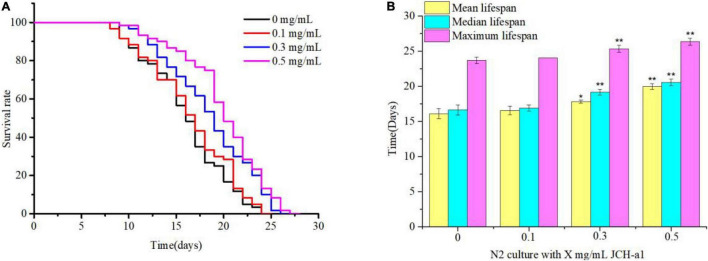
JCH-a1 can extend the lifespan of *C. elegans*. **(A)** At 20°C, *C. elegans* longevity was cultured at different concentrations of JCH-a1 and survival was monitored. **(B)** At 20°C, the mean lifespan, median lifespan and maximum lifespan of *C. elegans* were cultured at different concentrations of JCH-a1. * represents significant difference (*P* < 0.05), and ^**^ represents extremely significant difference (*P* < 0.01).

Lifespan represents the most intuitive evaluation index in the process of nematode aging. Synchronized *C. elegans* were fed the JCH-a1 (0.1, 0.3, 0.5 mg/mL). The results showed that the three concentrations of polysaccharides could prolong their lifespan compared with the control group (Figure 8). The high-dose group had a positive effect on delaying aging, with a maximum life span of 26.3 days and the average lifespan increased by 24.1%. The average lifespan of nematodes increased by 10.7% in the medium dose group and 2.8% in the low dose group. The results showed that under normal culture conditions, polysaccharides may delay the aging of wild-type worms.

### Lifespan Under Different Stressors

**FIGURE 9:**
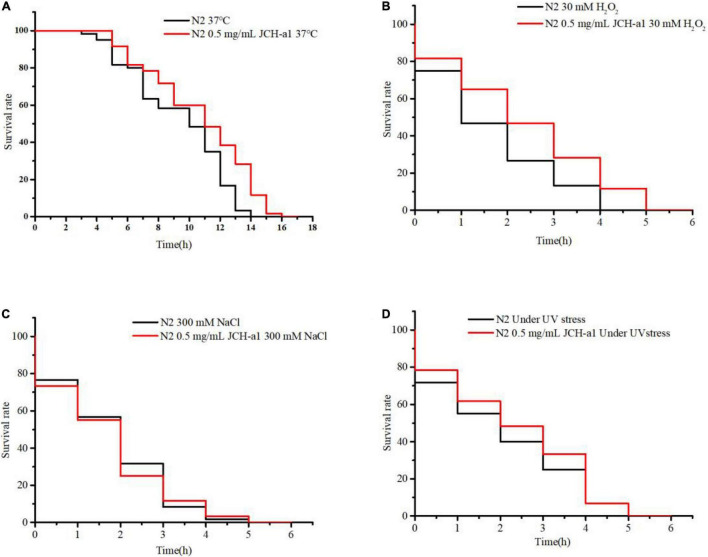
JCH-a1 improved the health of *C. elegans*. **(A)** Survival curves of N2 with or without JCH-a1 at 37°C for heat stress. **(B)** Survival of N2 cultured with or without JCH-a1 in S-buffer containing 30 mM H_2_O_2_ for oxidative stress. **(C)** Survival of N2 cultured with or without JCH-a1 in S-buffer containing 300 mM NaCl for osmotic stress. **(D)** Survival curves of N2 with or without JCH-a1 at UV conditions for UV stress.

Many studies have reported that antioxidants can prolong the lifespan of *C. elegans* by improving the ability of stress response (42). It can be seen from Figure 9 that the lifespan of N2 cultured with 0.5 mg/mL JCH-a1 under heat, oxidation and ultraviolet stress was longer than that of the control group, but it was basically the same as that of the control group under osmotic stress. In other words, JCH-a1 can improve the ability of nematodes to resist high temperature, enhance antioxidant response, and improve the ability to resist ultraviolet stress, but it has no effect on osmotic stress.

### The Effects of Antioxidant System

In order to study the effect of *C. cicadae* polysaccharide on antioxidant defense system, we measured the antioxidant enzyme activity and MDA content of nematodes (Table 1). In polysaccharides at different concentration groups, SOD, CAT, and GSH-Px activity increased significantly, which compared with the activity of the control group (*P* < *0.05*), and the activity was concentration-dependent. After pretreatment with three concentrations of *C. elegans*, SOD activity increased significantly by 5.0, 18.4, and 44.5% (*P* < 0.05). Similarly, compared with the untreated group, CAT activity increased by 11.6, 39.9, and 49.6%, respectively (*P* < *0.05*). The activities of GSH-Px increased by 5.0, 68.1, and 67.9, respectively (*P* < *0.05*). However, the MDA levels of low- dose-, medium-dose- and high-dose-treated *C. elegans* decreased significantly by 7.1, 23.9, and 46.2% compared with the treatment of the control group, (*P* < *0.05*). The results showed that the high dose group had the strongest ability to improve the activity of antioxidant enzymes. The results of enzyme activity and MDA showed that JCH-a1 had good antioxidant activity *in vivo*.

**TABLE 1:** Effect of the polysaccharide on the antioxidant enzyme activity and malondialdehyde content of *C. elegans*.

Treatment	Antioxidant enzyme activity^1^			MDA content^2^
	SOD	CAT	GSH-Px	
0 mg/mL	47.91 ± 0.42d	64.26 ± 1.48d	54.82 ± 0.65b	3.53 ± 0.18a
0.1 mg/mL	50.29 ± 0.79c	71.73 ± 1.88c	57.55 ± 0.74b	3.28 ± 0.07a
0.3 mg/mL	56.72 ± 0.19b	89.93 ± 3.34b	92.15 ± 1.77a	2.68 ± 0.06b
0.5 mg/mL	69.21 ± 0.88a	96.15 ± 2.72a	92.03 ± 1.63a	1.90 ± 0.09c

*The worms treated with JCH-a1. The antioxidant enzyme activity and MDA content of worms were measured. Mean with different letters in a row are significantly different (P < 0.05). The values shown are the means ± SD of 100 worms.*

*^1^SOD, CAT and GSH-Px U/mg protein.*

*^2^MDA nmol/mg protein.*

## Discussion

### Selection of Extraction Methods

Polysaccharides can be extracted in many ways, and the extraction rate of polysaccharides obtained by different methods and materials is also different. Thus, the best extraction process needs to be determined according to the specific situation (43). As mentioned in the introduction, UAEE has been used to extract various natural polysaccharides, but it has not been used in *C. cicadae*. In addition, the factors affecting the polysaccharide extraction yield include solvent properties, temperature, time, solid-liquid ratio, and ultrasonic power (44). Therefore, in order to evaluate the interaction between the extraction process parameters, the experimental design of UAEE in *C. cicadae* polysaccharide was carried out by box Behnken design software, and the effects of process parameters as well as their interaction on response variables were evaluated by RSM method. In addition, ultrasonic power is fixed to reduce the complexity of optimization operations caused by excessive process parameters. The addition of enzyme, ultrasonic temperature, ultrasonic time, and liquid-solid ratio were optimized in four factors and three levels. The results showed that under UAEE conditions, the extraction rate of polysaccharides was significantly higher than that of EAE and UAE. Wang et al. (45) also obtained higher yield of dandelion polysaccharide (14.05%) using UAEE than EAE (9.84%) and UAE (8.84%). Yang et al. (46) also attained higher pectin yield (31.1%) from sisal waste using UAEE compared with EAE (9.4%) and UAE (11.9%). These reports show that UAEE has great advantages in improving the yield of polysaccharides.

### Antioxidant Activity

The excessive production of reactive oxygen free radicals will destroy the structure of biological macromolecules and cause serious oxidative damage to the body (47). Preventing oxidative aging can reduce its damage to human body by reducing ROS. Studies have shown that bioactive polysaccharides from different natural sources play an important role in scavenging free radicals and can be used as new antioxidants (48). This study showed that *C. cicadae* polysaccharide had strong scavenging activities against DPPH, ABTS, •OH, and •O^2–^ free radicals. In animal organism, GSH-Px, SOD, and CAT are important components of endogenous antioxidant system (49). It can catalyze the decomposition of ROS groups such as •O^2–^, •OH and H_2_O_2_ in cells, which is conducive to maintaining the balance of the body and delaying the aging of *C. elegans* (50). MDA is one of the end products of lipid peroxidation induced by reactive oxygen species (ROS), which can be used as the end point of oxidative stress evaluation (51). Thus, *C. cicadae* polysaccharide JCH-a1 may prolong the lifespan of *C. elegans* by scavenging excess reactive oxygen species, enhancing enzyme activity, and reducing MDA level. Therefore, the polysaccharide of *C. cicadae* has a good antioxidant effect and can be used as a new dietary supplement to delay the aging process.

### Inhibitory Effect on α-Glucosidase

Carbohydrates, like starch and glycogen, are important nutrients that can provide the body with the necessary energy for normal life activities (52). These long-chain carbohydrates are hydrolyzed and absorbed, which can cause postprandial hyperglycemia. α-glucosidase decompose polysaccharides into oligosaccharides, so reducing its activity can control postprandial hyperglycemia in patients with type 2 diabetes (53). Nature polysaccharides can effectively inhibit α-glucosidase activity (54). Our study also showed that polysaccharide JCH-a1 can act as a mixed inhibitor of α-glucosidase and inhibit its activity. So, the JCH-a1 can reduce postprandial blood glucose level to a certain extent and prevent diabetes and its complications.

### Evaluation of Structure-Activity Relationship

Generally, the activity of polysaccharides is affected by the chemical characteristics of polysaccharides, such as molecular weight, monosaccharide composition, polysaccharide conformation, and the way of glycosidic bonds (55). The optimum molecular weight range for the bioactivity of polysaccharides from different sources is different. The high molecular weight polysaccharide (16.9 kDa) of *Athyrium multidentatum* showed strong antioxidant activity (56). The components with lower molecular weight (36.7 kDa) of *Inonotus Obliquus* polysaccharides had stronger inhibition of α-glucosidase (57). However, the low molecular weight polysaccharide (15.3 kDa) of wild *Armillaria mellea* had higher free radical scavenging and metal chelating ability (58). This study found that the molecular weight of *C. cicadae* polysaccharide was 60.7 kDa, which also has good antioxidant and hypoglycemic activities. Thus, it can be speculated that the effect of polysaccharide molecular weight on activity is not clear.

Monosaccharide composition can affect the chain structure and higher structure of polysaccharides, and the higher structure is an important factor affecting the activity of polysaccharides (59). It was found that polysaccharides containing monosaccharides such as arabinose, mannose, galactose, and xylose usually have good antioxidant activity (60). Therefore, the good antioxidant activity of JCH-a1 may be attributed to the presence of mannose, glucose, and galactose.

Previous studies have shown that polysaccharides with β-configuration have higher activity (61). However, the JCH-a1 was mainly α-configurated and had strong biological activity. Similarly, α-configurated polysaccharide in *Grifola Frondosa* had immune function (62). Hence, there is no clear conclusion on the relationship between heterocephalic carbon configuration and polysaccharide bioactivity.

The bioactivity of polysaccharides is not only related to its primary structure, but also closely related to the higher conformation of sugar chain. It is generally believed that polysaccharides with regular spatial conformation and triple helix have higher activity. The triple helix configuration is the active spatial structure of polysaccharides. Surenjav et al. (63) found that natural trichelix *Lentinus Edodes* had obvious antitumor biological activity, while the antitumor biological activity of polysaccharide with single flexible chain decreased significantly. Consequently, *C. cicadae* polysaccharide has good antioxidant and hypoglycemic activities, which may be related to its triple helix structure.

## Conclusion

In conclusion, this study found that UAEE could significantly improve the extraction efficiency of polysaccharides from *C. cicadas*. Furthermore, purified component JCH-a1 may be a supplement dietary bioactive ingredient for antioxidant and hypoglycemic activity. Finally, it also provides a basis for the establishment of structure-activity relationship of *C. cicadas* polysaccharides.

## Data Availability Statement

The original contributions presented in this study are included in the article/Supplementary Material, further inquiries can be directed to the corresponding author/s.

## Author Contributions

ZT and WL designed and performed the experiment. YuC, SF, YQ, HoC, and YL analyzed the data and drew diagrams and tables. YX drafted the work. HuC supervised and administrated the work. CD, TB, QL, YiC, and HY revised it critically for important content. All authors contributed to the article and approved the submitted version.

## Conflict of Interest

The authors declare that the research was conducted in the absence of any commercial or financial relationships that could be construed as a potential conflict of interest.

## Publisher’s Note

All claims expressed in this article are solely those of the authors and do not necessarily represent those of their affiliated organizations, or those of the publisher, the editors and the reviewers. Any product that may be evaluated in this article, or claim that may be made by its manufacturer, is not guaranteed or endorsed by the publisher.
