# Supplementary material for: Extraction, Purification, Physicochemical Properties, and Activity of a New Polysaccharide From Cordyceps cicadae
Source: Front Nutr. 2022 Jun 9;9:911310. doi: 10.3389/fnut.2022.911310 (PMC9218675; doi:10.3389/fnut.2022.911310)
Supplement: Supplementary file 2 [file Data_Sheet_2.docx]

Table S1. Independent variables and their levels used in the BBD^a^.

| Factor | Symbol | Level | | |
| --- | --- | --- | --- | --- |
|  |  | −1 | 0 | 1 |
| Enzymatic addition | A | 0.4 | 0.6 | 0.8 |
| Ultrasonic temperature | B | 50 | 60 | 70 |
| Ultrasonic time | C | 10 | 20 | 30 |
| Liquid-solid ratio | D | 35 | 45 | 55 |

BBD: Box–Behnken design. A: Addition (%), B: Temperature (℃), C: Time (min), D: Liquid-solid ratio (mL/g)

Table S2 BBD arrangement for the extraction of JCH and the observed responses for extraction yield.

| Test number | A (%) | B (℃) | C (min) | D (mL/g). | Extraction yield (%) |
| --- | --- | --- | --- | --- | --- |
| 1 | 0.6 | 60 | 30 | 55 | 3.07 |
| 2 | 0.4 | 60 | 20 | 55 | 3.58 |
| 3 | 0.6 | 50 | 30 | 45 | 3.25 |
| 4 | 0.8 | 60 | 10 | 45 | 2.95 |
| 5 | 0.6 | 60 | 20 | 45 | 3.72 |
| 6 | 0.6 | 50 | 10 | 45 | 2.68 |
| 7 | 0.6 | 70 | 20 | 35 | 2.75 |
| 8 | 0.4 | 70 | 20 | 45 | 3.45 |
| 9 | 0.8 | 50 | 20 | 45 | 3.03 |
| 10 | 0.4 | 60 | 10 | 45 | 3.26 |
| 11 | 0.8 | 60 | 30 | 45 | 2.55 |
| 12 | 0.4 | 60 | 20 | 35 | 2.65 |
| 13 | 0.6 | 60 | 20 | 45 | 3.45 |
| 14 | 0.8 | 70 | 20 | 45 | 2.74 |
| 15 | 0.6 | 60 | 30 | 35 | 2.59 |
| 16 | 0.6 | 60 | 20 | 45 | 3.59 |
| 17 | 0.8 | 60 | 20 | 35 | 2.94 |
| 18 | 0.6 | 60 | 10 | 35 | 2.84 |
| 19 | 0.6 | 70 | 10 | 45 | 3.46 |
| 20 | 0.6 | 60 | 20 | 45 | 3.76 |
| 21 | 0.6 | 60 | 10 | 55 | 3.44 |
| 22 | 0.4 | 50 | 20 | 45 | 3.36 |
| 23 | 0.4 | 60 | 30 | 45 | 2.97 |
| 24 | 0.6 | 60 | 20 | 45 | 3.71 |
| 25 | 0.6 | 70 | 20 | 55 | 3.29 |
| 26 | 0.6 | 50 | 20 | 55 | 3.29 |
| 27 | 0.6 | 70 | 30 | 45 | 2.55 |
| 28 | 0.8 | 60 | 20 | 55 | 2.66 |
| 29 | 0.6 | 50 | 20 | 35 | 2.89 |

The values of extraction yield are mean (n = 3). BBD: Box–Behnken design.

Table S3. Analysis of variance for extraction yield from BBD experiments.

| Source | Sum of squares | df | Mean square | F-value | P-value | significance |
| --- | --- | --- | --- | --- | --- | --- |
| Model | 4.02 | 14 | 0.2875 | 22.69 | < 0.0001 | significant |
| A | 0.4800 | 1 | 0.4800 | 37.89 | < 0.0001 | *** |
| B | 0.0056 | 1 | 0.0056 | 0.4447 | 0.5157 |  |
| C | 0.2269 | 1 | 0.2269 | 17.91 | 0.0008 | *** |
| D | 0.5941 | 1 | 0.5941 | 46.89 | < 0.0001 | *** |
| AB | 0.0361 | 1 | 0.0361 | 2.85 | 0.1135 |  |
| AC | 0.0030 | 1 | 0.0030 | 0.2388 | 0.6327 |  |
| AD | 0.3660 | 1 | 0.3660 | 28.89 | < 0.0001 | *** |
| BC | 0.5476 | 1 | 0.5476 | 43.22 | < 0.0001 | *** |
| BD | 0.0049 | 1 | 0.0049 | 0.3868 | 0.5440 |  |
| CD | 0.0036 | 1 | 0.0036 | 0.2842 | 0.6023 |  |
| A2 | 0.6457 | 1 | 0.6457 | 50.97 | < 0.0001 | *** |
| B2 | 0.3752 | 1 | 0.3752 | 29.61 | < 0.0001 | *** |
| C2 | 0.9453 | 1 | 0.9453 | 74.62 | < 0.0001 | *** |
| D2 | 0.7247 | 1 | 0.7247 | 57.20 | < 0.0001 | *** |
| Residual | 0.1774 | 14 | 0.0127 |  |  |  |
| Lack of Fit | 0.1132 | 10 | 0.0113 | 0.7064 | 0.7021 | NS |
| Pure Error | 0.0641 | 4 | 0.0160 |  |  |  |
| Cor Total | 4.20 | 28 |  |  |  |  |
| Pred R-Squared: 0.8209 | | Adj R-Squared: 0.9156 | | Adeq Precisior: 15.1535 | | R^2^ = 0.9578 |

*P < 0.05, **P < 0.01, ***P < 0.001, NS: not significant. BBD: Box–Behnken design.

Table S4 Comparison of the [polysaccharide](https://www.sciencedirect.com/topics/biochemistry-genetics-and-molecular-biology/polysaccharide) yields by different extraction methods.

| Extraction methods | Extraction conditions | | | | | The yield of JCH (%) |
| --- | --- | --- | --- | --- | --- | --- |
|  | A | B | C | D | ultrasonic power |  |
| UAEE | 0.71 | 60 | 18 | 46 | 240 | 3.66 ± 0.87% |
| EAE | 0.71 | 60 | 18 | 46 | – | 1.73 ±0.69% |
| UAE | – | 60 | 18 | 46 | 240 | 1.54 ± 0.46% |

A: Addition (%), B: temperature (℃), C: Time (min), D: Liquid-solid ratio (mL/g).


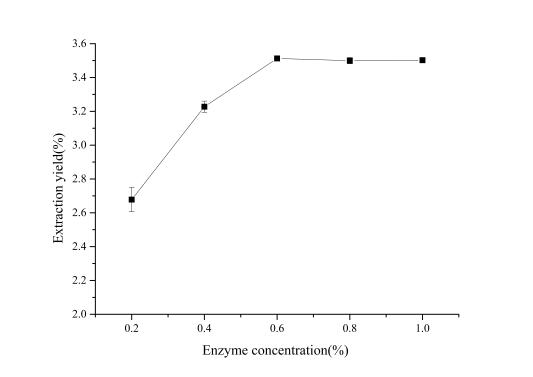

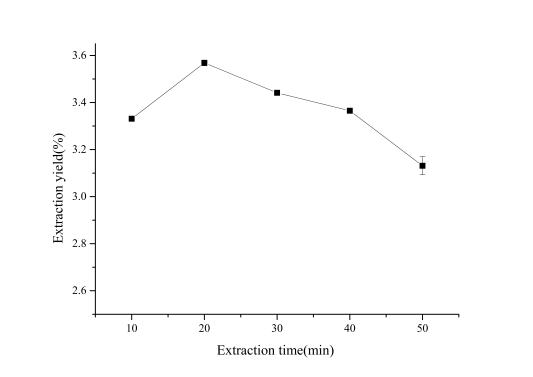

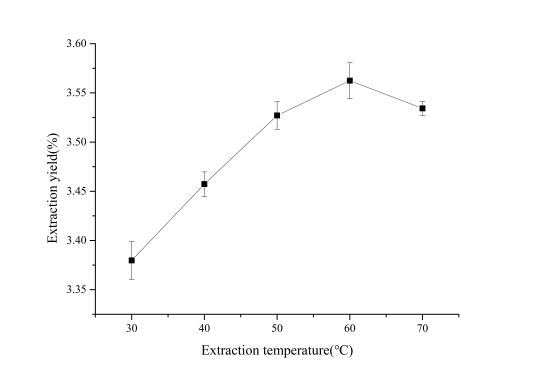

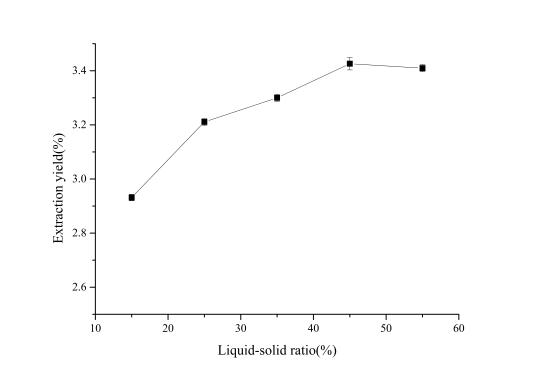


D

C

B

A

**Fig. S1.** Effects of different experimental conditions on the yield of JCH. (A) Enzymatic addition; (B) Ultrasonic temperature; (C) Ultrasonic time; (D) Liquid-solid ratio.


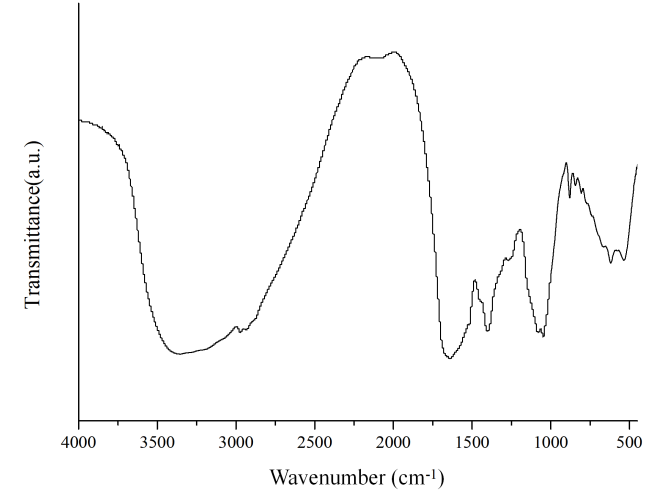


**Fig. S2.** FT-IR spectrogram of JCH-a1.





**Fig. S3.** DSC thermogram of JCH-a1.

**Fig. S4.** XRD spectra of JCH-a1.


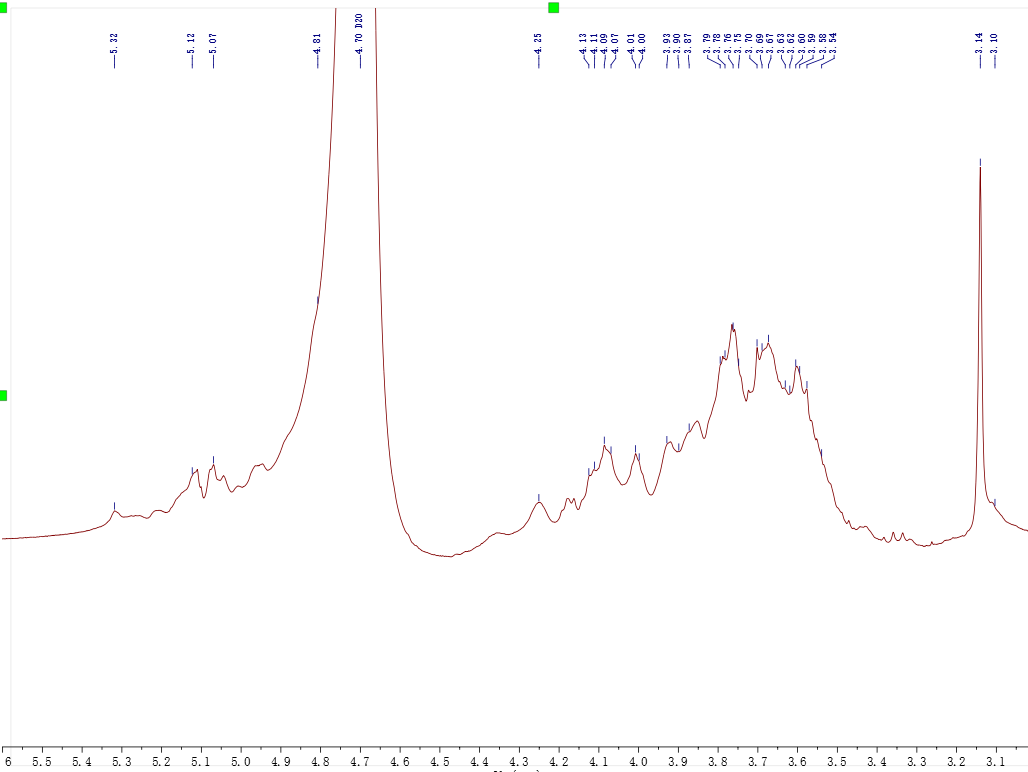


A


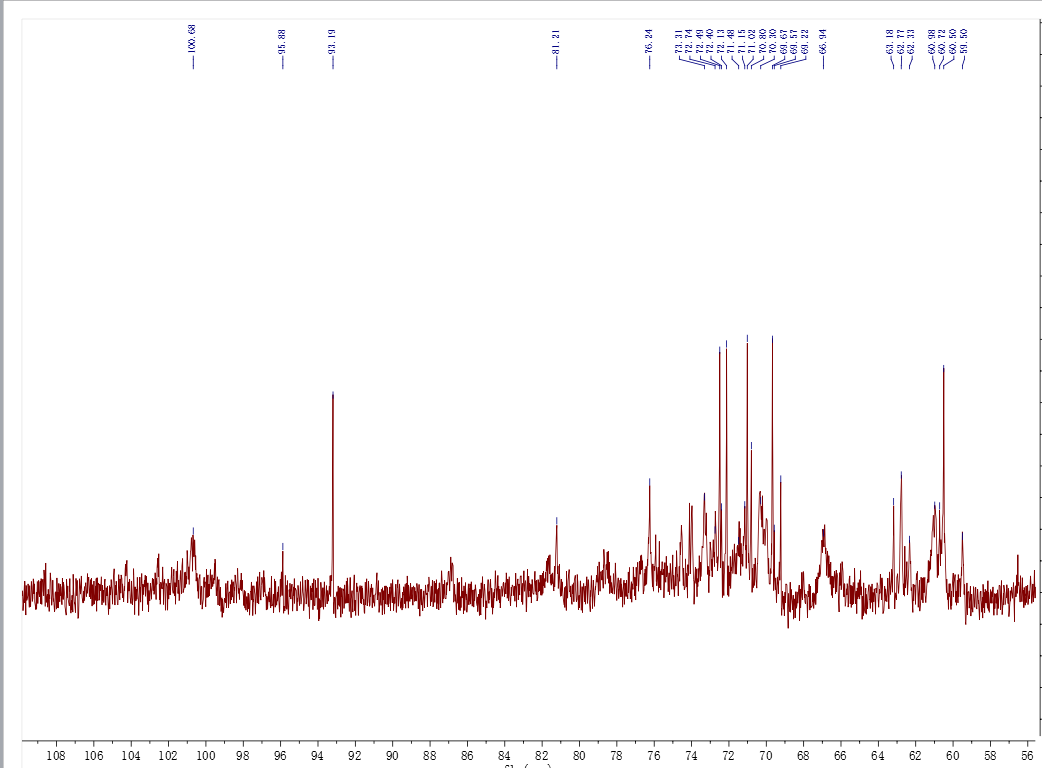


B

**Fig. S5.** NMR spectra of JCH-a1. (A) ^1^H NMR spectra; (B)^13^C NMR spectra.


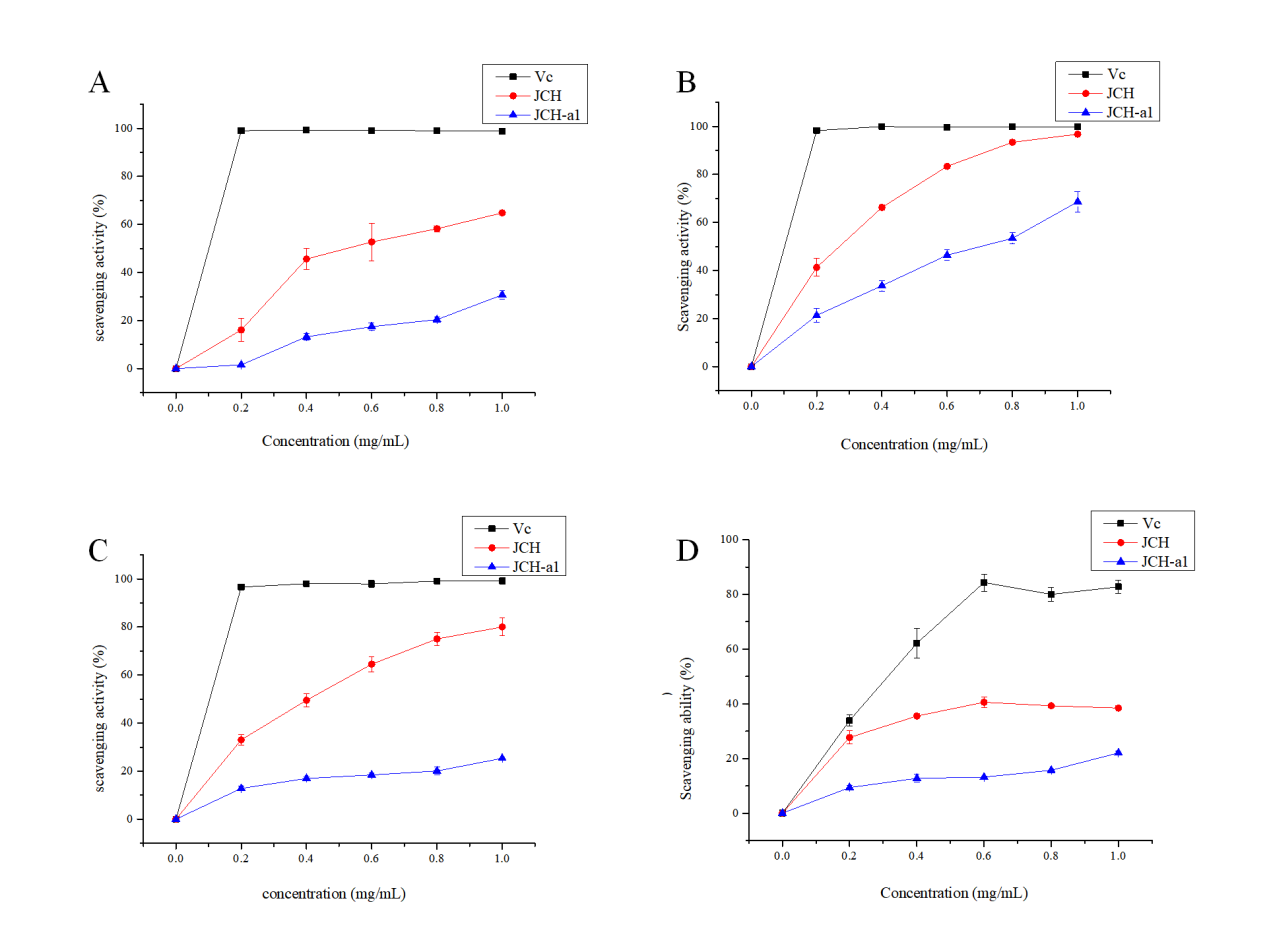


**Fig. S6.** Antioxidant activity of JCH, JCH-a1 and V_C_. (A) DPPH radical scavenging activity. (B) ABTS radical scavenging activity. (C) Hydroxyl radical scavenging activity. (D) Superoxide radical scavenging activity.
